# Comparing feedback from faculty interactions and virtual assessment software in the development of psychomotor skills in preclinical fixed prosthodontics

**DOI:** 10.1002/cre2.129

**Published:** 2018-09-14

**Authors:** Ramtin Sadid‐Zadeh, Elizabeth H. D'Angelo, Joseph Gambacorta

**Affiliations:** ^1^ Department of Restorative Dentistry University at Buffalo School of Dental Medicine New York

**Keywords:** Compare software, dental education, fixed prosthodontics, immediate feedback, virtual assessment software

## Abstract

The purpose of this study was to determine the effectiveness of virtual assessment software as a means of immediate feedback for preclinical fixed prosthodontics course. The subjects of the study were second year dental students with no previous training in fixed prosthodontics. Nine students participated in the study. Participants completed 2 days of didactic training focused on the principles of tooth preparation and the use of intraoral scanners and virtual assessment software. Didactic training was followed by 12 sessions of practical exercises. Students were randomly assigned to one of three groups for training in the preparation of tooth no. 46 for a complete cast crown. Students received feedback from (a) faculty interaction only; (b) interactions with both faculty and virtual assessment software; or (c) interactions with only virtual assessment software. During Sessions 5, 10, and 12, students prepared tooth no. 46 for complete cast crown independently and without any immediate feedback to simulate a practical exam. Prepared teeth were collected at Sessions 5, 10, and 12, and two blinded faculty members assessed the teeth following an established rubric. Results from Session 12 showed that preparations that one of three students and two of three students respectively for student–faculty interaction and student–faculty–Compare software interaction groups did not meet acceptable standards. However, the students in student–Compare software interaction group generated acceptable preparations at week 12. These data suggest that immediate feedback via virtual assessment software may be as effective as one‐on‐one faculty instruction for dental students in fixed prosthodontics.

## 1 INTRODUCTION

Preclinical dental instruction is conducted in a simulated clinical environment in which students develop psychomotor skills by practicing on a dental mannequin. The goal of this simulated environment is to create a clinic‐like setting for students to prepare teeth. Preclinical teaching environments can be divided into four types: (a) laboratories with mannequin heads mounted on metal rods, (b) contemporary simulation clinics, (c) clinical simulations conducted in actual treatment clinics, and (d) virtual reality simulation (VRS) clinics (Jasinevicius, Landers, Nelson, & Urbankova, 2004).

The VRS setting enables training students to be provided with immediate feedback regarding their tooth preparation skills. Although an early study found no significant difference in final grades between students who used VRS in addition to traditional training and those with only traditional training, more recent studies have suggested that the use of VRS can improve student performance in preclinical simulations (Buchanan, 2004; LeBlanc, Urbankova, Hadavi, & Lichtenthal, 2004; Urbankova, 2010). For example, early stage psychomotor skills were shown to be improved by the use of VRS paired with conventional faculty–student interactions (LeBlanc et al., 2004; Urbankova, 2010). Similarly, a study by Yasukawa (2009) showed that students who interacted solely with VRS for immediate feedback received a significantly lower grade on their practical exams compared with those who interacted with faculty and VRS. In contrast, Kikuchi, Ikeda, and Araki (2013) found that when students used VRS to learn to prepare a metal ceramic crown, interactions with faculty had no effect on student performance.

Based on these results, the effectiveness of VRS compared with conventional dental student training approaches is not completely clear. It is generally agreed that VRS show great promise in dentistry, but the conventional simulation environment is still considered to have a place in facilitating interactions with faculty who can provide critical feedback (Quinn, Keogh, McDonald, & Hussey, 2003; Wierinck, Puttemans, & Steenberghe, 2006). However, even with VRS, faculty can still play an important role in helping students to gain experience by providing feedback (Quinn et al., 2003).

Accurate and consistent objective assessment of dental students in the preclinical setting is critical for proper psychomotor skill development. Studies have shown that faculty members are not able to assess students consistently, even when the same faculty evaluates the same work on different occasions (Fuller, 1972; Lilley, Bruggen Cate, Holloway, Holt, & Start, 1986; Salvendy, Hinton, Ferguson, & Cunningham, 1973). In an attempt to overcome this subjectivity in assessment, use of clear standards and well‐defined assessment forms has been recommended (Knight, 1997). Faculty members can be trained and calibrated to evaluate students using standardized assessment forms to reduce subjectivity. However, additional variables, such as part‐time versus full‐time status, teaching experience, education level, and dedication to teaching, can influence faculty calibration, resulting in inconsistent feedback and impaired student psychomotor development (Knight, 1973).

In addition to providing immediate feedback, VRS evaluations are also objective and consistent, factors that positively influence student psychomotor skill development. In an attempt to remove the “human factor” from assessment of dental student performance, virtual assessment software (Compare software, Planmeca/E4D Technologies, Richardson, TX, USA) has been developed for use in preclinical fixed prosthodontics. Software‐generated comparison percentages calculated by virtual comparison of student preparations versus a standard preparation have been suggested as a reliable and consistent method of student assessment (Renne et al., 2013). However, to date, no correlation has been found between faculty assessments of student work and comparison percentages (Callan, Haywood, Cooper, Furness, & Looney, 2015).

Similar to VRS, Compare software may be used for immediate feedback. Advantages of using Compare software versus VRS for immediate feedback are (a) a customized rotary instrument is not needed; (b) a specific setup for capturing the motion is not needed; (c) lower cost; (d) students get trained to use the intraoral scanner as part of using Compare software. No study has evaluated the influence of using Compare software for immediate feedback on psychomotor development in fixed dental prosthodontics.

The purpose of this study was to determine whether Compare software (Planmeca/E4D Technologies) provides appropriate feedback for students working to develop psychomotor skills needed for tooth preparation in fixed prosthodontics. The specific aim of this study was to evaluate the effects of student interactions with faculty alone, Compare software alone, or a combination of the two on student performance in preparation of a first mandibular molar (tooth no. 46) for complete cast crown.

## 2 MATERIALS AND METHODS

This study was conducted in accordance with the Declaration of Helsinki (1964) as revised in Venice in 1983 and was approved by the Institutional Review Board of the University at Buffalo (IRB No. FWA00008824). Informed written consent was obtained from all students prior to initiation of the study. Each participant reserved the right to withdraw from the study at any time.

Second year students at the University at Buffalo School of Dental Medicine (UB SDM) were invited to participate in this study with the goal of recruiting 36 students. Invited students had successfully passed their first year technical courses, but they had not enrolled in a fixed prosthodontics preclinical course prior to the study. Fifteen students signed the consent form to participate, but six students withdrew prior to the start of the study.

The study was conducted during summer break when no classes were being held at UB SDM. Students completed four sessions of didactic training, followed by 12 sessions of practical exercises. The duration of each session was 4 hr. Students were not permitted to work after dedicated hours. During the four didactic training sessions, students listened to presentations about supplies, principles of tooth preparation, step‐by‐step preparation of tooth no. 46 for complete cast crown, and assessment forms. In addition, students were provided with hands‐on training in the use of intraoral scanners (Planmeca Corp.) and Compare software (Planmeca/E4D Technologies). Training on Compare software was focused on (a) a three‐dimensional visual comparison of student preparations versus a standard preparation; (b) measured differences between the superimposed images of student and standard preparations using a slice plane tool and a distance tool; (c) quantification of total occlusal convergence (TOC) using a Loma Linda TOC guide on mesio‐distal and bucco‐lingual slices of student preparations. Students were then provided with the presentations, a video showing preparation of tooth no. 46 for complete cast crown in a preclinical setting, a three‐dimensional view of a standard tooth prepared for complete cast crown, and a physical model of the standard preparation.

In addition, students were provided with a high‐speed handpiece, required diamond cutting tools (850, 6878K‐016, 8878K‐016, 6847K‐016, and 8847K‐016 diamond burs; Brasseler USA; Savannah, GA), a diamond‐cleaning stone (NTI®; Kerr Dental), a dental mirror, an explorer, and a periodontal probe (Hu‐friedy), polyvinyl siloxane (Lab‐Putty; Coltene/Whaledent AG), sharpies, a Loma Linda TOC guide; and a dentiform (Series Model 200, Nissan Dental Products) with the associated teeth. In addition, a virtual model of the standard preparation was provided to students in the student–Compare software interaction (SCI) and student–faculty–Compare software interaction (SFCI) groups for use in the Compare software.

At the end of the didactic training sessions, students were randomly divided into three groups, each three students. The randomization was done using Microsoft Excel. To learn the skills required for preparation of tooth no. 46 for a complete cast crown, each group was assigned to one of the following teaching methodologies: (a) student–faculty interaction (SFI), where students only interacted with faculty members; (b) SCI, where students only interacted with Compare software; and (c) SFCI, where students interacted with faculty in conjunction with the use of Compare software. The students were assigned identifying numbers that started with SFI, SCI, or SFCI, depending on their assigned group. The group identifier was followed by the student's initials; however, to protect the identities of the students, we have identified them by the letter A, B, or C (Example: CSI‐A) in this article. One student from the SCI group left the study at Session 3 of the training due to a lack of interest in the use of Compare software for training, leaving the SCI group with two students.

After randomization of students to the three groups, two previous preclinical course directors for the Dental Anatomy and Direct Restoration courses scored the participants' psychomotor skills using a scale ranging from 1 to 3. Scoring was based on how quickly the students developed psychomotor skills in previous courses, where 1 was defined as rapidly, by the end of the first third of the semester; 2 was defined as moderately, during the second third of the semester; and 3 was defined as slowly, in the last third of the semester. The practical exams grades were considered as an indicator for the psychomotor development.

All students completed 12 consecutive training sessions at the UB SDM simulation facility, where students prepared tooth no. 46 on a dentiform with an oral cavity cover (Series Model 200, Nissan Dental Products, Japan) mounted on a pole. The length of each session was 4 hr, and students were not allowed to practice out of these sessions. The faculty member who taught the didactic portion of the training supervised the preclinical sessions. All students could refer to the didactic documents provided to them at any time. During the preclinical sessions, students in the SFI and SFCI groups interacted with faculty member and received immediate feedback. The only difference between the SFI and the SFCI group was that the SFCI group had access to Compare software for immediate feedback. The only feedback source for the SCI group was Compare software, which was used to superimpose their preparation against a standard preparation. The SCI group did not receive any instructions beyond the documents provided and the basic information received during didactic sessions. Answers to technical questions about their equipment or to procedural questions about intraoral scanning or Compare software comprised the only instruction this group received from faculty members.

Each student received teeth nos. 45, 46, and 47 at each session, and by the completion of each session, he or she should have finalized preparation for at least one tooth no. 30 for complete cast crown. Prepared teeth were collected at the end of each session in an envelope labeled with the student's identification number, the session number, and the date.

Session 1 was the baseline, even though students spent 4 hr to prepare the tooth and they could communicate with their teaching tools depending on their assigned group. Sessions 5, 10, and 12 were considered to be practical exam days. Students did not interact with faculty or with Compare software during these 2.5‐hr sessions. At the end of the study, two blinded and calibrated faculty members evaluated the prepared teeth from Sessions 1, 5, 10, and 12. Preparations were evaluated for the damage of adjacent teeth, amount of occlusal reduction, TOC, presence or absence of undercut, contour of the axial wall, axial wall height, finish line location, finish line quality, and finish of the preparation (Table 1). Each preparation was marked as excellent (E), standard (S), or standard not met (N) for each criterion. Preparations scored as N for TOC and finish line quality were considered to exhibit the presence of undercut and unsupported enamel, respectively. In cases where two faculty members disagreed on a criterion, a third blinded and calibrated faculty member made the final decision. Due to the small sample size, the data gathered from this study were compared descriptively.

**Table 1:** Criteria assessed and assessment methods for evaluation of teeth prepared during practical exams

Assessed criterion	Assessment method
Damage to adjacent teeth	Visual inspectionTactile sensation with explorer
Amount of occlusal reduction	Reduction guidePeriodontal probe
Total occlusal reduction (TOC)–undercut	Loma Linda TOC guideVisual inspection
Contour of the axial wall	Visual inspection
Axial wall height	Periodontal probe
Finish line location	Periodontal probe
Finish line quality–unsupported enamel	Visual inspection6878K‐016 diamond bur
Finish of the preparation	Visual inspection

## 3 RESULTS

Table 2 presents psychomotor skill scores evaluated by two preclinical course directors for dental anatomy and direct restoration courses, median of psychomotor score for each student and for each study group. Students in the SCFI group had a higher median compared with students in the SFI and SCI groups.

**Table 2:** Initial psychomotor scores for each subject

Subject	Dental anatomy	Direct restoration	Median per subject	Media per study group
SFI‐A	1	2	1.5	2
SFI‐B	3	1	2	
SFI‐C	2	2	2	
SFCI‐A	3	3	3	2.5
SFCI‐B	3	2	2.5	
SFCI‐C	2	2	2	
SCI‐A	2	3	2.5	2
SCI‐B	2	1	1.5	

**Figure 1:**
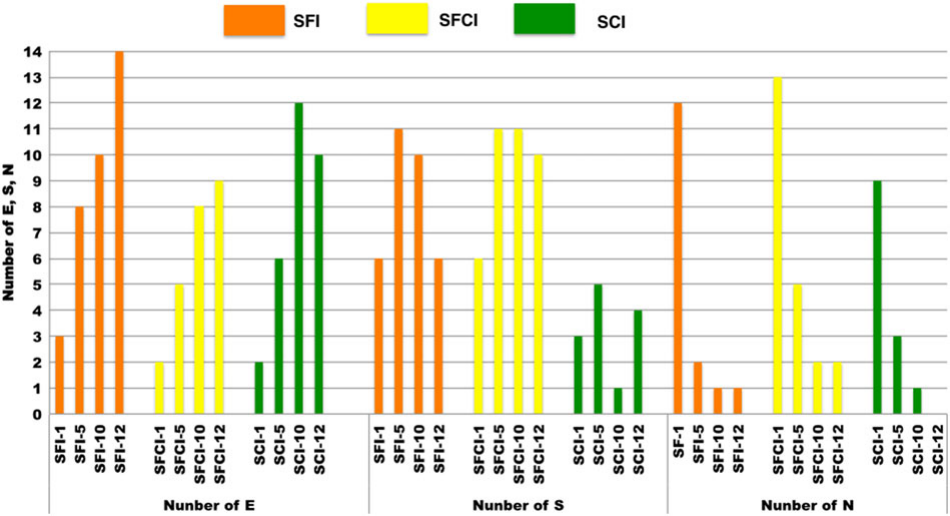
Total number of E, S, and N scores received following assessment form at Sessions 1, 5, 10, and 12 for each instructional group. SCI: student–Compare software interaction; SFCI: student–faculty–Compare software interaction; SFI: student–faculty interaction

Figure 1 shows total number of E, S, and N scores following assessment form (Table 1) by group at Sessions 1, 5, 10 and 12. Comparison of scores from these sessions reveals a trend showing improvement over time for each group, evident as an increase in the number of Es and a decrease in the number of Ns received from Sessions 1 to 12. A total of 24 preparations were collected from practical exams (Session 1 was not practical exam). Seventeen errors were observed in 24 preparations, the most common of which was presence of undercut, which was observed in seven preparations (29%). The remainder of the errors included unsupported enamel (in three preparations), finish of the preparation (in two preparations), finish line width (in two preparations), the amount of occlusal reduction (in two preparations), and contour of the preparation (in one preparation).

**Figure 2:**
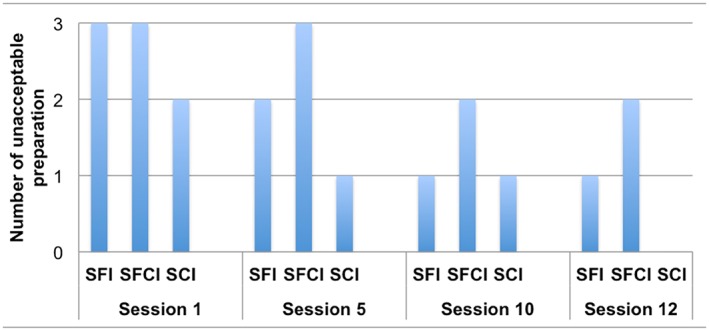
Number of unacceptable preparations generated by each group at Sessions 1, 5, 10, and 12. SCI: student–Compare software interaction; SFCI: student–faculty–Compare software interaction; SFI: student–faculty interaction

Figure 2 depicts the number of unacceptable preparations for each group at the baseline and Sessions 5, 10, and 12. At the baseline, none of the students prepared an acceptable tooth. At Session 5, one preparation each from the SFI and SCI groups and three preparations from the SFCI group were considered to be unacceptable. At Session 10, four prepared teeth were unacceptable, one from each of the SFI and SCI groups and two from the SCFI group. At Session 12, two teeth from the SFCI group and one tooth from the SFI group did not meet the requirements for an acceptable preparation. By the last session, all of the students in the SCI group generated an acceptable preparation. Considering total preparations in the practical exam Sessions 5, 10, and 12, 4 out of 9 (44%), 7 out of 9 (77%), and 2 out of 6 (33%) of the preparations were not acceptable in the SFI, SFCI, and SCI groups, respectively.

**Figure 3:**
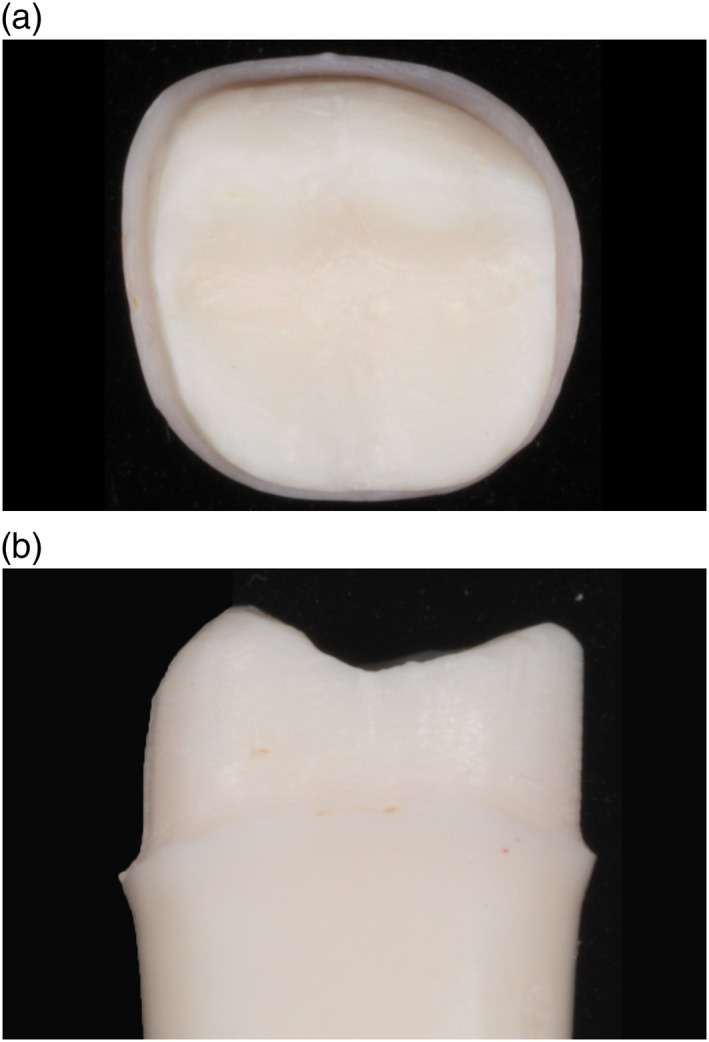
Unacceptable tooth prepared by student SFI‐B at Session 12, (a) occlusal view. Unacceptable tooth prepared by student SFI‐B at Session 12, (b) mesial view

**Figure 4:**
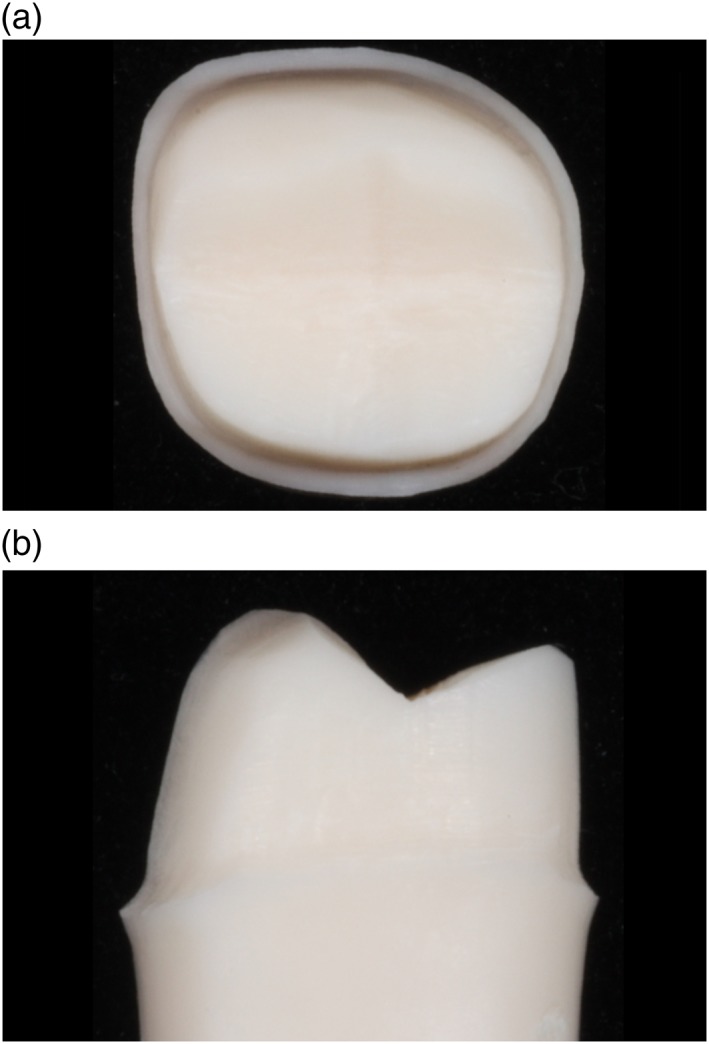
Acceptable tooth prepared by student SFI‐C at Session 12, (a) occlusal view. Acceptable tooth prepared by student SFI‐C at Session 12, (b) mesial view

**Figure 5:**
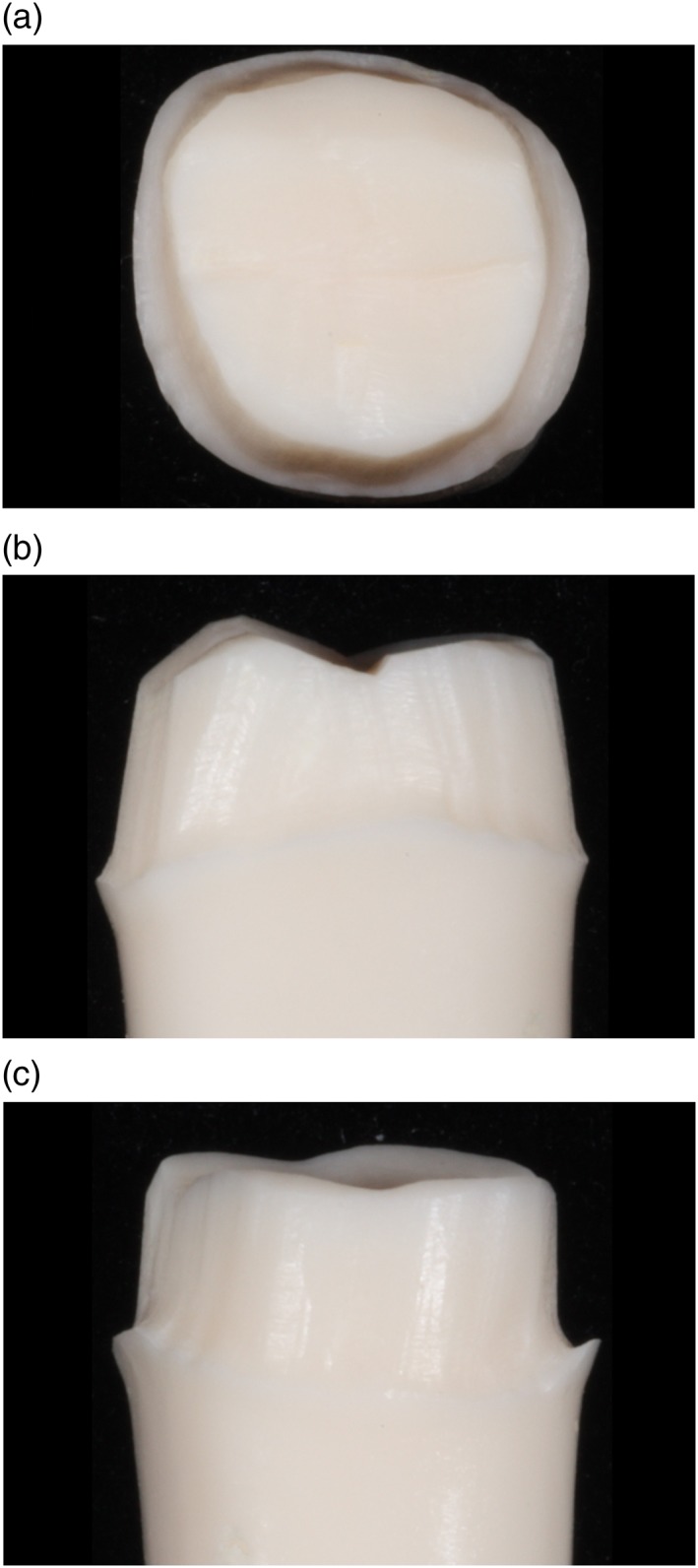
Unacceptable tooth prepared by student SFCI‐A at Session 12, (a) occlusal view. Unacceptable tooth prepared by student SFCI‐A at Session 12, (b) mesial view. Unacceptable tooth prepared by student SFCI‐A at Session 12, (c) lingual view

**Figure 6:**
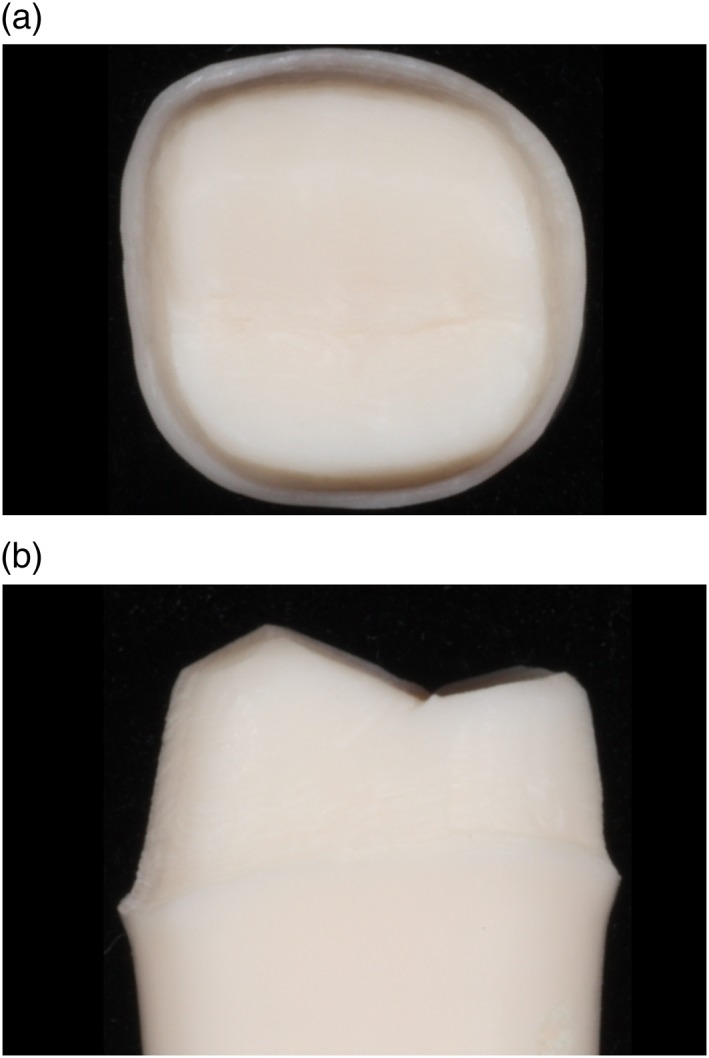
Acceptable tooth prepared by student SFCI‐C at Session 12, (a) occlusal view. Acceptable tooth prepared by student SFCI‐C at Session 12, (b) mesial view

**Figure 7:**
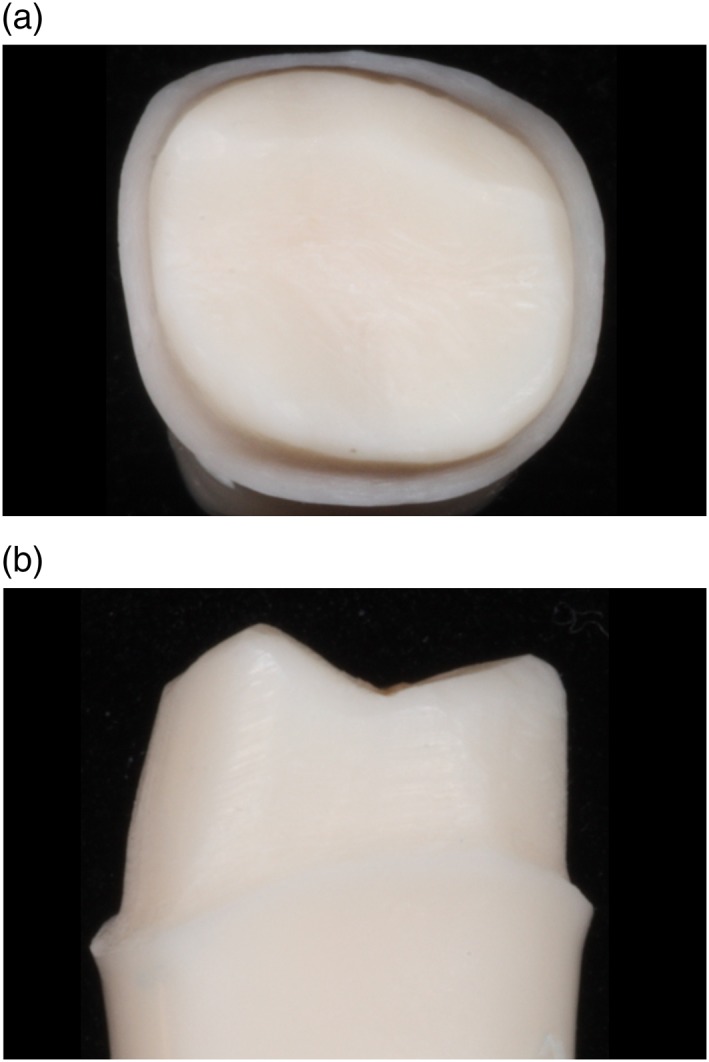
Acceptable tooth prepared by student SCI‐A at Session 12, (a) occlusal view. Acceptable tooth prepared by student SCI‐A at Session 12, (b) mesial view

Figures 3, 4, 5, 6, 7 show an example of acceptable and unacceptable tooth for each group at Session 12. Except for the presence of undercut on the tooth prepared by student SFI‐B, the quality of the preparations for the SFI and SCI groups were better than that of the SFCI group. Es comprised 58% (14 of 24 criteria) and 62% (10 of 16 criteria) of the scores received by the SFI and SCI groups, respectively. In comparison, the SFCI group received only nine Es out of 24 criteria (37%) at Session 12.

## 4 DISCUSSION

The study limitations included (a) eight students participated in the study; (b) grade as the motivational factor was not considered for the study; and (c) it was performed in 12 consecutive sessions. However, the study was designed to recruit 36 students and the students were motivated by payment upon the completion of the study, following IRB approval. In addition, the summer break period for dental students was limited and the educational facility to be used for the study was scarce during the academic calendar, so it was not applicable for the authors to run the study in a period of a full semester.

Differences in psychomotor skill development observed in this study could be related to the progress in the previous courses. Students in the SFCI group received the slowest psychomotor skill development scores at the initial evaluation. This trend was also observed in scores from practical exams on Sessions 5, 10, and 12, in which students in the SFCI group had a higher number of unacceptable tooth preparations compared with the other groups. Students in the SFI and SFCI groups who received a psychomotor skill development score of 3 from one or both previous course directors did not generate a satisfactory tooth preparation for full‐coverage restoration at Session 12 (SFI‐B, SFCI‐A, SFCI‐B). In contrast, the student in the SCI group who received a psychomotor skill development score of 3 from the direct restoration course director performed satisfactorily (SCI‐A). However, this difference in performance may not have resulted from using the Compare software for immediate feedback, because students in the SFCI group also had access to Compare software. This difference could also have been related to motivation, defined as “effort” allocated to tasks (Yeo & Neal, 2004). Motivation has been shown to influence the relationship between ability and performance (Kanfer & Ackerman, 1989). Students who did not perform satisfactorily may not have had sufficient motivation to put forth the effort required to master preparation of the tooth.

Seventeen errors were observed for the 24 teeth prepared during the practical exams. The most common errors were undercut, unsupported enamel, and finish of the preparation, which accounted for 70.6% (12 of 17) of the errors. Even though the performance of the students in the SCI group was acceptable at Session 12, the majority of errors observed were ones for which Compare software could not provide immediate feedback to the students.

A previous study found that when students interacted solely with VRS for immediate feedback, they received lower grades on their practical exams compared with students who interacted with faculty along with VRS (Yasukawa, 2009). Due to the limited number of students, no statistical analysis was performed for this study; however, the descriptive comparison suggests that the SCI group performed better than the SFCI group. It is important to note that this difference might be due to the higher initial psychomotor skill scores in SFCI group. Additionally, the results may have been different if the sample size were larger.

In another attempt, Jasinevicius et al. (2004) evaluated the influence of VRS on the quality of tooth preparation by students. They compared the preparation for a full coverage restoration after training students by VRS and SFI. Their finding showed that VRS group had a higher average score compared with the SFI group; however, there was no significant difference between the two groups. Similarly, this study showed SCI group performed better than SFI group. However, the difference may have been insignificant, if the sample size was larger.

Percentage of unacceptable preparation was 44%, 77%, and 33% in the SFI, SFCI, and SCI groups, respectively. Although the number of students was very small in this study, the students in the SCI group performed better than those in the other groups. Thus, the results of this study suggest that some students may benefit from use of Compare software as an immediate feedback mechanism to develop their psychomotor skills in the absence of faculty feedback.

A shortage of faculty has been characterized as the most critical challenge confronting dental schools today (John et al., 2011). The results of this study show that Compare software may serve as a useful means of immediate feedback for some students. Thus, implementation of Compare software could be used to compensate for insufficient numbers of dental school faculty. In addition, supplementation with software could also give the faculty the opportunity to focus on students who need more one‐on‐one assistance in order to properly develop their psychomotor skills for tooth preparation.

## 5 CONCLUSIONS

Under limitation of this study, it may be concluded that Compare software may be as effective as faculty for providing immediate feedback regarding a full coverage tooth preparation for specific students. Our findings indicate that the use of Compare software holds promise for the training of future dentists. However, additional research using a larger number of study subjects is necessary to draw a definitive conclusion about the complete reliability of the software in the dental education setting.

## CONFLICT OF INTEREST

None declared.
